# Exploring the Relationship Between Mandibular Morphology, Dental Eruption, and Chronological Age in Modern Human Juveniles Through Geometric Morphometrics

**DOI:** 10.1002/ajpa.70155

**Published:** 2025-11-12

**Authors:** Tannistha Chakraborty, Julie Arnaud, Costantino Buzi

**Affiliations:** ^1^ Department of Anthropology University of Central Florida Orlando Florida USA; ^2^ Department of Humanities University of Ferrara Ferrara Italy; ^3^ UMR 7194—HNHP, Muséum National d’Histoire naturelle of Paris Paris France; ^4^ Institut Català de Paleoecologia Humana i Evolució Social (IPHES‐CERCA) Tarragona Spain; ^5^ Departament d'Història i Història de l'Art Universitat Rovira i Virgili Tarragona Spain; ^6^ DFG Centre of Advanced Studies ‘Words, Bones, Genes, Tools’ University of Tübingen Tübingen Germany; ^7^ Department of Physics and Geology University of Perugia Perugia Italy

**Keywords:** age assessment, bioarchaeology, dental development, mandibular growth, virtual anthropology

## Abstract

**Objective:**

This study investigated how mandibular morphological shape and dental eruption patterns reflect chronological age in modern human juveniles, using geometric morphometrics. The aim was to assess their variation, covariation, and the accuracy of classifying individuals into age‐based groups using combined shape information.

**Materials and Methods:**

Computed tomography scans from a sex‐balanced sample of 48 individuals, aged 4 to 13 years, sourced from the New Mexico Decedent Image Database, were used to generate 3D models of mandibular bodies and permanent dentition (incisors, canines, premolars, and first molars). Mandibular and dental morphology were analyzed using 3D landmark‐based methods. Generalized Procrustes Analysis and Principal Component Analysis (PCA) assessed shape variability. Two‐block partial least squares analysis evaluated covariation and linear regression tested the age estimation protocol.

**Results:**

PCA of combined proxies revealed distinct morphological changes in the mandible corresponding to different phases of dental eruption. In contrast, analyses of separated proxies exhibited high variability, limiting their reliability. The combined configuration demonstrated a strong association between mandibular shape and dental eruption patterns, aligning closely with the chronological ages of individuals in the sample.

**Discussion:**

This study highlights the utility of integrating mandibular shape variation with dental eruption as an effective approach for capturing consistent morphological changes during growth. The combined proxies exhibit strong statistical relations with chronological age and reliably identify developmental change (shape change) even within narrow age brackets of 3–6 months, providing a foundation for the development of a standardized age estimation protocol.

## 1 Introduction

Assessing the chronological age of skeletal remains is a fundamental objective in bioarchaeology, particularly in constructing the biological profile of unidentified individuals. Age estimation methods typically rely on indicators of biological maturity, that is the progression from an immature to a skeletally and reproductively mature adult state (Himes 2004). This process is a key feature of human juvenility and is reflected in hard tissues through the prolonged course of dental development and continuous skeletal growth, offering a temporal framework for estimating age at death in juvenile remains (Scheuer and Black 2000; Šešelj 2013). While standard methods often rely on a single indicator, such as dental formation or skeletal ossification, researchers have increasingly argued that no single maturity marker sufficiently captures the complex, multidimensional nature of human growth and development (Simpson et al. 1991; Bogin 1999; Dean 2000; Franklin 2010; T. M. Smith 2013; Hillson 2014; Mânica 2018). Consequently, reliance on individual indicators can introduce significant inaccuracies such as imprecise age range estimations, tendencies toward overestimation or underestimation of true age, lack of standardization in sample selection, and inconsistencies in calculating, expressing, and interpreting errors when validating methods (Bedford et al. 1993; Saunders et al. 1993; Bocquet‐Appel and Masset 1996; Maber et al. 2006; Cunha et al. 2009; Liversidge et al. 2010; Merritt 2013; Valsecchi et al. 2019).

In response, several studies have attempted to improve age estimation by examining the relationship between dental development and skeletal ossification (Liliequist and Lundberg 1971; Ubelaker 1987; Lampl and Johnston 1996; Scheuer and Black 2000; Sciulli 2007; Šešelj 2013, 2017; Bittencourt et al. 2018; Corron et al. 2018; Cunningham 2019; Smith et al. 2021; Coquerelle et al. 2011, 2013). These comparative studies have contributed significantly to our understanding of growth coordination; however, they also underscore the need for further investigation into cranial elements, particularly the facial skeleton, developmentally, and functionally more directly associated with the dentition (Freidline et al. 2017, 2025; Quintino et al. 2025). In this context, the mandible offers a valuable opportunity to examine the integration of skeletal and dental maturation, due to its role in both supporting the dentition and reflecting age‐related morphological change. The mandible is one of the most variable bones in the skull; yet, due to its high preservation in archeological contexts, it serves as one of the most reliable skeletal elements for reconstructing the biological profile of an individual. Dental eruption takes place after crown formation of teeth is complete and the teeth move through the alveolar bone until reaching occlusion (Scheuer and Black 2000). Both mandibular morphology and dental eruption are highly dependent upon chronological age and have been used as standard data for estimating age, investigating the ontogeny of a population or species (B. H. Smith 1991; Buikstra and Ubelaker 1994; Braga et al. 2004; Mellion et al. 2013).

The present study employed an exploratory application of Virtual Anthropology (Weber et al. 2001) and Geometric Morphometrics to examine the relationship between dental eruption patterns, mandibular shape variation and chronological age in modern human juveniles, on age and sex‐balanced predetermined samples. The primary objective of the study was to assess the morphological variability of the combined proxies across different phases of dental eruption, and to evaluate whether integrating mandibular morphology with dental eruption patterns could improve age estimation accuracy compared to using each data point independently. Specifically, the study seeks to: (i) identify age‐group‐specific trends during dental eruption phases; (ii) examine the relationship between tooth eruption and mandibular shape; (iii) evaluate the efficacy of combining both proxies for age estimation.

## 2 Materials and Methods

### 2.1 Study Sample

The sample analyzed in this study consisted of 48 individuals (25 male and 23 female) aged between 4 and 13 years old, collected from the New Mexico Decedent Image Database (Edgar et al. 2020). Since the analysis focused on the period of mixed dentition, spanning from crown completion to the eruption of permanent teeth except the second and third molar (White et al. 2011), two distinct intervals were used for sample selection to capture the two phases of permanent dentition development (Scheuer and Black 2000). For individuals aged 4 to 6 years, a sampling interval of three months was maintained, with one male and one female selected for each interval. For individuals aged 6 to 13 years, a 6‐month sampling interval was used. A longer interval was deemed sufficient for this age range, as dental and mandibular changes tend to slow down at this stage compared to the rapid developmental changes occurring in the preceding one (Franchi et al. 2001). It is important to note that the sample collection was based on cross‐sectional radiographic data, and any individuals with pathological abnormalities or signs of trauma were excluded.

To better understand the morphological changes occurring during the different stages of the permanent dental eruption, the sample was divided into five distinct groups, from unerupted to completely erupted permanent teeth. The detailed definition of the groups is reported in Table 1. The list of the samples, separated into the groups and the dental stage of the individuals in the sample is reported in Table S1.

**TABLE 1:** Groups used in the study and their definitions.

Group	*N*.	Age range	Description
G1	4F 4M	4–5 years (48–61 months)	Permanent teeth unerupted: molar, incisor, canine, and premolar are below the alveolar level.
G2	5F 7M	4.4–7.5 years (53–91 months)	Molar and incisor partial eruption: M1 and I1 or I2 are at the alveolar level or halfway between the alveolar bone and the occlusal level. Canine and premolars are below the alveolar level.
G3	5F 7M	7–10.5 years (84–126 months)	Molar and incisor complete eruption: M1 and both I1 and I2 are at the occlusal level. Canine and premolars are below the alveolar level.
G4	3F 5M	8.2–12.3 years (99–148 months)	Premolar, canine partial eruption: M1 and both I1 and I2 are at the occlusal level. C and P3 or 4 are at the alveolar level or halfway between the alveolar bone and the occlusal level.
G5	6F 2M	11–12.9 years (134–155 months)	Complete eruption: molars, premolars, canine, and incisors are at the occlusal level.

*Note:* The number of individuals per group (*N*.) is divided by the number of females (F) and males (M).

### 2.2 Data Preparation

The Computed Tomography scan images of each individual's skull were segmented to generate separated 3D models of the mandible and the mandibular dentition. The procedure was performed by a single operator (TC), using the open‐access software 3D Slicer (http://www.slicer.org, Fedorov et al. 2012).

### 2.3 Geometric Morphometrics

Two distinct landmark configurations were defined (including type I, type II and type III landmarks, Bookstein 1992), for the mandible (n. 30, both type I and II) and the mandibular permanent dentition (n.12, type II and type III), by using the 3D Slicer Markup Tool (Fedorov et al. 2012).

**FIGURE 1:**
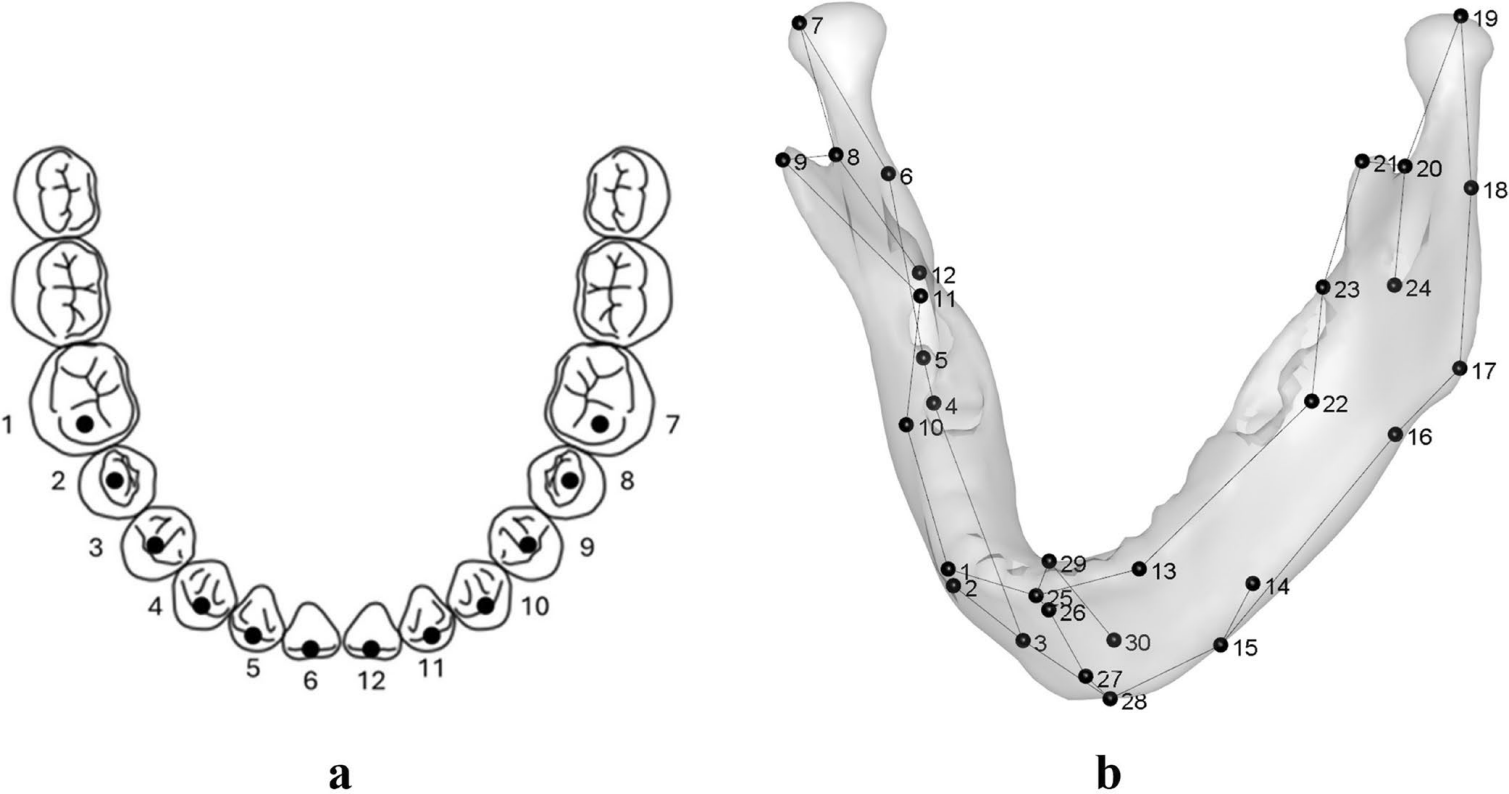
Landmark configurations. (a) Teeth configuration (T); (b) Mandibular configuration (M).

For the anterior dentition (Table S2), landmarks were placed on the tip of the cusp for the canine (C) and at the midpoint of the incisal margin for the incisors (I1, I2). For the posterior dentition, specific buccal cusps were used (Figure 1a). The mandibular landmark configuration was adapted from Bastir et al. (2007) and Rosas and Bastir (2004), and is presented in Figure 1b and Table S3. The landmark sampling was performed by a single operator (TC). Both landmarks' configurations were standardized for size and position through a General Procrustes Analysis (GPA, Rohlf and Slice 1990).

We tested the intraobserver error by acquiring the landmarks on a subsample of 10 individuals three times each; we then performed a Multivariate ANOVA on the Procrustes coordinates by using the function *procD.lm* of the package *geomorph* (v. 4.0.9, Adams and Otárola‐Castillo 2013). The results obtained returned significant differences among individuals (*p*‐value < 0.001; *R* ^2^ = 0.997) and no significant differences between the repetitions (*p*‐value = 0.149; *R* ^2^ = 0.0004).

We extracted the landmark coordinates separately (teeth, T; mandible, M) and then combined them (teeth + mandible, TM) to develop a single configuration. The processing of the landmark data was done by using the R statistical environment (R Core Team 2022, version 4.4.2).

To address the primary objective of the study, assessing whether the combination of mandibular morphology and dental eruption patterns can improve chronological age estimation, we employed a set of complementary multivariate analyses, each selected to answer a specific aspect of the research question.

Principal Component Analysis (PCA) was performed on the Procrustes coordinates to highlight morphological variation across individuals in a reduced number of dimensions. PCA was used to explore whether the combined configuration (TM) provides clearer clustering of individuals by age group compared to mandibular or dental data alone. If the combined proxies better capture age‐related variation, we expected to observe tighter and more distinct clusters corresponding to the predefined age groups in the PCA space.

Partial Least Squares (PLS) analysis, using a two‐block approach, was applied to assess the degree of covariation between mandibular morphology and dental eruption patterns (Rohlf and Corti 2000). A strong covariation would suggest that changes in one structure are systematically associated with changes in the other during growth, supporting the idea that integrating both proxies could enhance age estimation accuracy.

Linear regression was conducted to test the relationship between each proxy (mandibular morphology, dental eruption pattern, and their combination) and chronological age. We expected that if one or both proxies are good predictors of age, the models would show a significant relationship with age, with the combined configuration yielding a higher explanatory power (e.g., higher *R* ^2^) than either proxy used alone.

Finally, to investigate potential size‐related shape changes (allometry), we performed linear regressions of the first principal components against the centroid size of each individual. This allowed us to assess the extent to which shape variation was driven by growth‐related size differences.

All analyses and visualizations were carried out in R, using the following packages: *Morpho* (v. 2.12; Schlager 2017), *Rvcg* (v. 0.24; Schlager 2017), *Arothron* (v. 2.0.5; Profico et al. 2021), *rgl* (v. 1.3.16; Murdoch and Adler 2025), *geomorph* (v. 4.0.9; Adams and Otárola‐Castillo 2013), and *ggplot2* (v. 3.5.1; Wickham 2011).

## 3 Results

### 3.1 Combined Configuration

**FIGURE 2:**
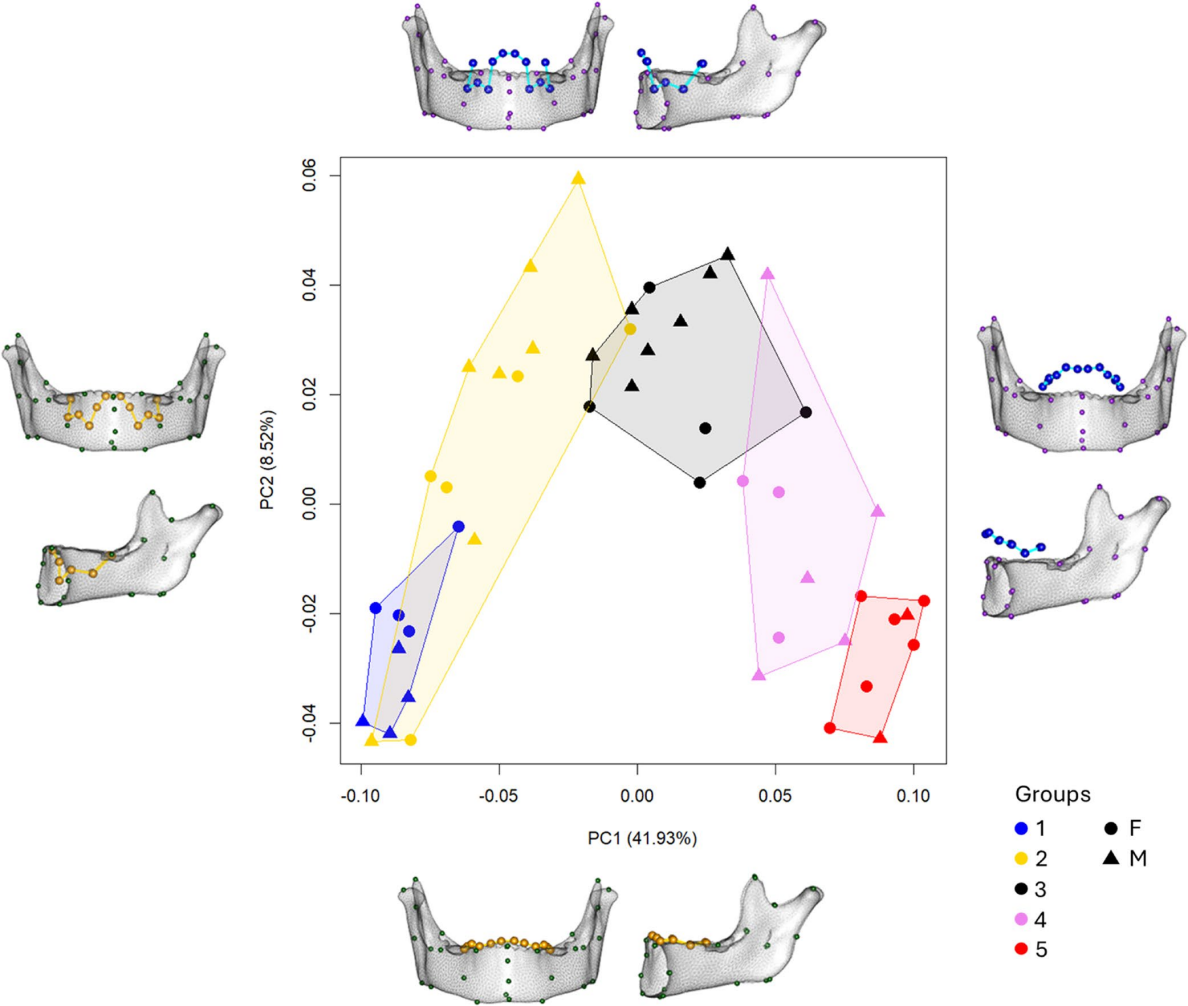
Principal Component 1 vs. Principal Component 2 of combined configuration (TM) and shape variations (frontal and lateral view), along the correspondent axis (G1: 48–61 months; G2: 53–91 months; G3: 84–126 months; G4: 99–148 months; G5: 134–155 months).

Figure 2 shows the results of the PCA performed on the Procrustes coordinates of the TM configuration. The first two PCs represent 41.93% (PC1) and 8.52% (PC2) of total shape variance, respectively. The shape variations in Figure 2 highlight the changes occurring along the two PCs: along PC1, the dentition shows vertical displacement of all the teeth, shifting from below the alveolar level (minimum values of PC1) to above the alveolar level (maximum values of PC1), thus indicating the transition from all unerupted to fully erupted teeth. Along PC2 it is possible to identify the changes in the relative height of dentition in the morphospace, with all teeth at the same level (minimum values of PC2) and the stages of mixed dentition, with the maximum difference corresponding to the maximum values of PC2. These correspond to a stage in which certain teeth, such as the first molars, and central and lateral incisors, have fully erupted, while others like canines and premolars are still below the alveolar level. While the shape variations along PC2 explain a limited amount of the sample variability, along PC1 we observe a series of consistent changes: (i) decrease in the mandibular angle resulting in a more vertical alignment of the condyle process, (ii) prognathism of the mental symphysis, (iii) elongation of the coronoid process, (iv) decrease in the angle of the mandibular notch, (v) reduction of the bigonion breadth, resulting in a less divergent ramus body, (vi) posterior shift of the mental foramen, (vii) increase in the length of the alveolar body. These changes occur in correspondence with the development of the dentition.

**FIGURE 3:**
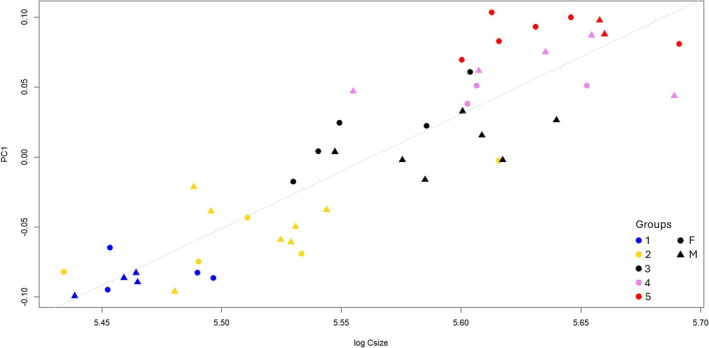
Regression of Principal Component 1 TM configuration against the log‐transformed centroid size, *R* ^2^ = 0.8025, *p* < 2.2e−16. (G1: 48–61 months; G2: 53–91 months; G3: 84–126 months; G4: 99–148 months; G5: 134–155 months).

Looking at the groups' position in the morphospace (Figure 2), we can observe along PC1 a sequence (from G1 to G5) with slight overlapping between the clusters (except for G5). Such important variability on PC1 can be easily explained by allometric effects, as presented in Figure 3, where PC1 is plotted against the log‐transformed centroid sizes. Along PC2, G1 and G5 fall along the lower values compared to G2–4. There is a notable inter‐individual variability, with G1 and G5 showing a smaller internal variability, while G2 seems to represent the highest one. We computed the variance by group for the first two PCs to quantify the patterns observed, and the results, reported in Table S4, confirm the highest variance for G2 and G3 (PC1) and G2 and G4 (PC2).

### 3.2 Separate Configurations

**FIGURE 4:**
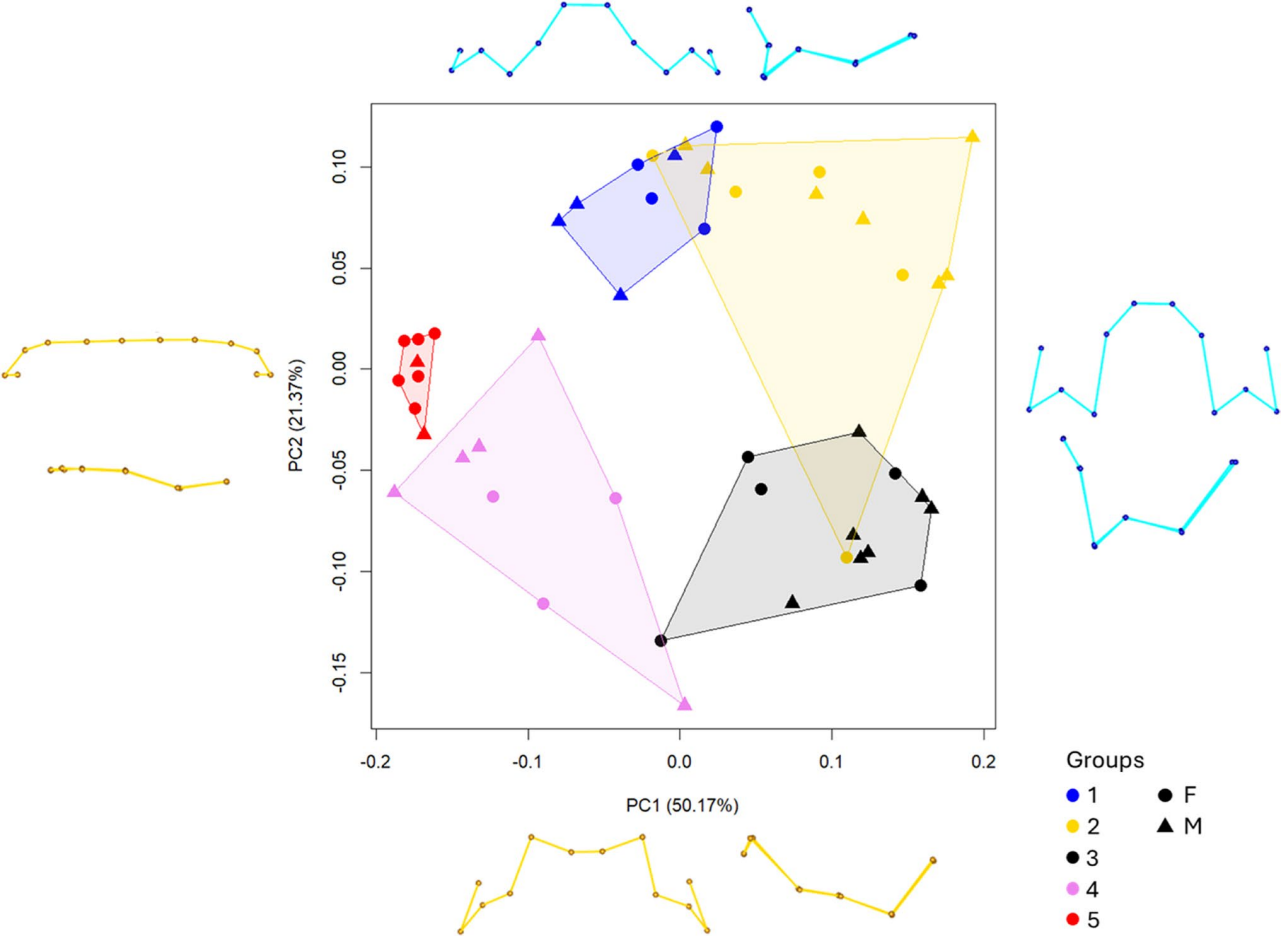
Principal Component 1 vs. Principal Component 2 of T configuration and shape variations, shown in frontal (top) and left lateral (bottom) views, along the correspondent axis. Yellow, minimum values; Cyan, maximum values (G1: 48–61 months; G2: 53–91 months; G3: 84–126 months; G4: 99–148 months; G5: 134–155 months).

**FIGURE 5:**
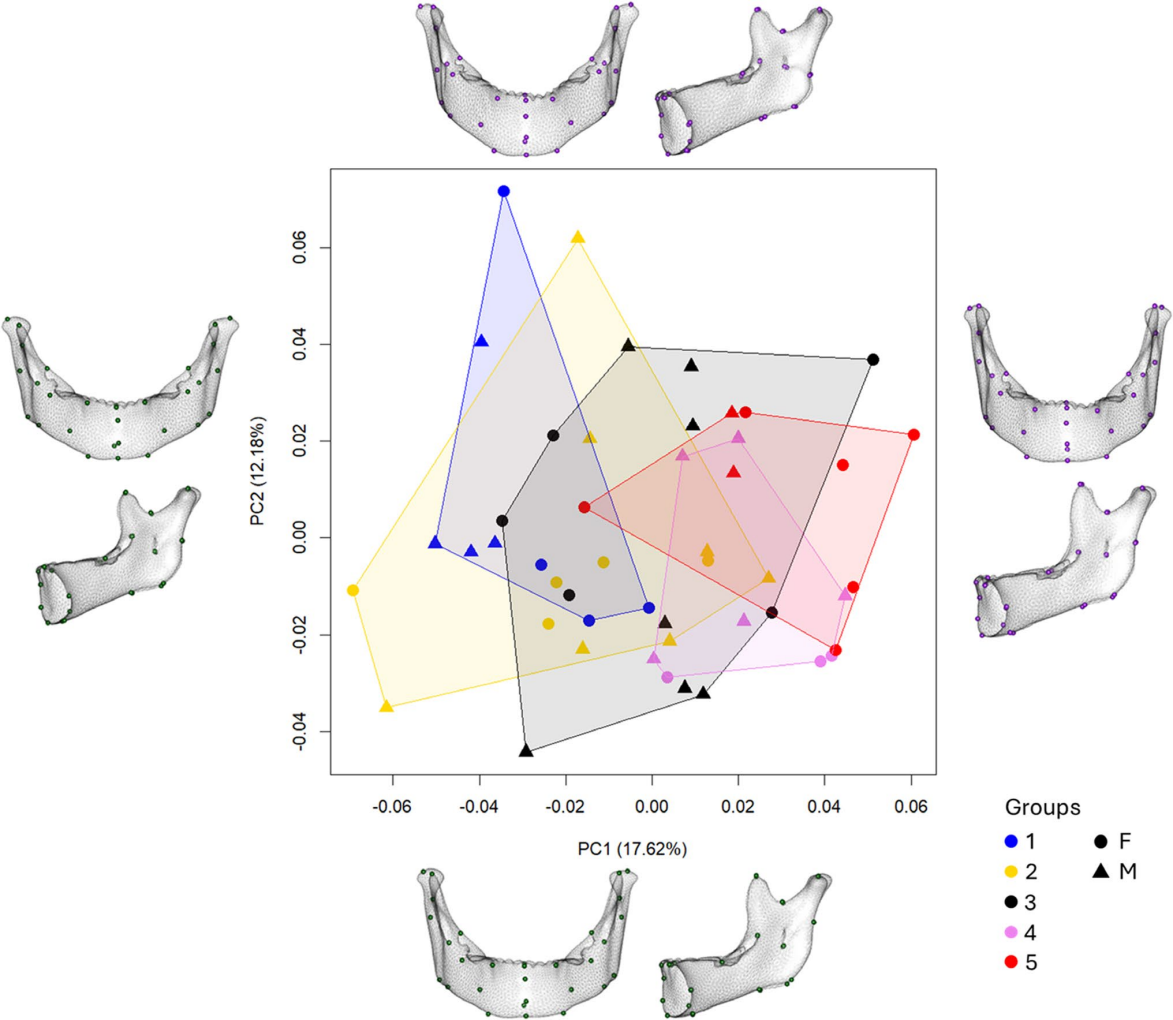
Principal Component 1 vs. Principal Component 2 of M configuration and shape variations (frontal and lateral view), along the correspondent axis. Green, minimum values; violet: maximum values (G1: 48–61 months; G2: 53–91 months; G3: 84–126 months; G4: 99–148 months; G5: 134–155 months).

A PCA of each separate configuration, T and M, was performed. The results, depicted in Figure 4 (T) and Figure 5 (M), illustrate the performance of each configuration individually, highlighting the group distribution and the morphological variations they undergo during growth. In addition, we compared the results of T and M separately with those of the TM configuration.

The scatterplot of T configuration (Figure 4) shows, along both PCs, a notable inter‐individual variability for the mixed‐dentition groups (i.e., G2–4) and a reduced internal variability for G1 and G5, which appear to be the group more strictly clustered. For the M configuration (Figure 5), there is no sensible difference between the shape variation along the PC1 and the same component analyzed for the TM configuration. On the other hand, along PC2, the shape variation shows a visible increase in the length range of the superior alveolar process and a decrease in the bigonial angle, with a reduction in the flexion of the ramus. In the morphospace (M configuration, Figure 5), all the groups overlap, and inter‐individual variability seems to be higher than that visible for T and TM configurations.

On the other hand, the T configuration (Figure 4) shows less overlap between the groups if compared to the M configuration. This is evidently due to the classification being based on the stage of dental eruption. Here, the groups (T) are centrally clustered, with slight overlap (except for G5), rather than having a sequential distribution as in Figure 2.

### 3.3 Two‐Block Partial Least Squares

**FIGURE 6:**
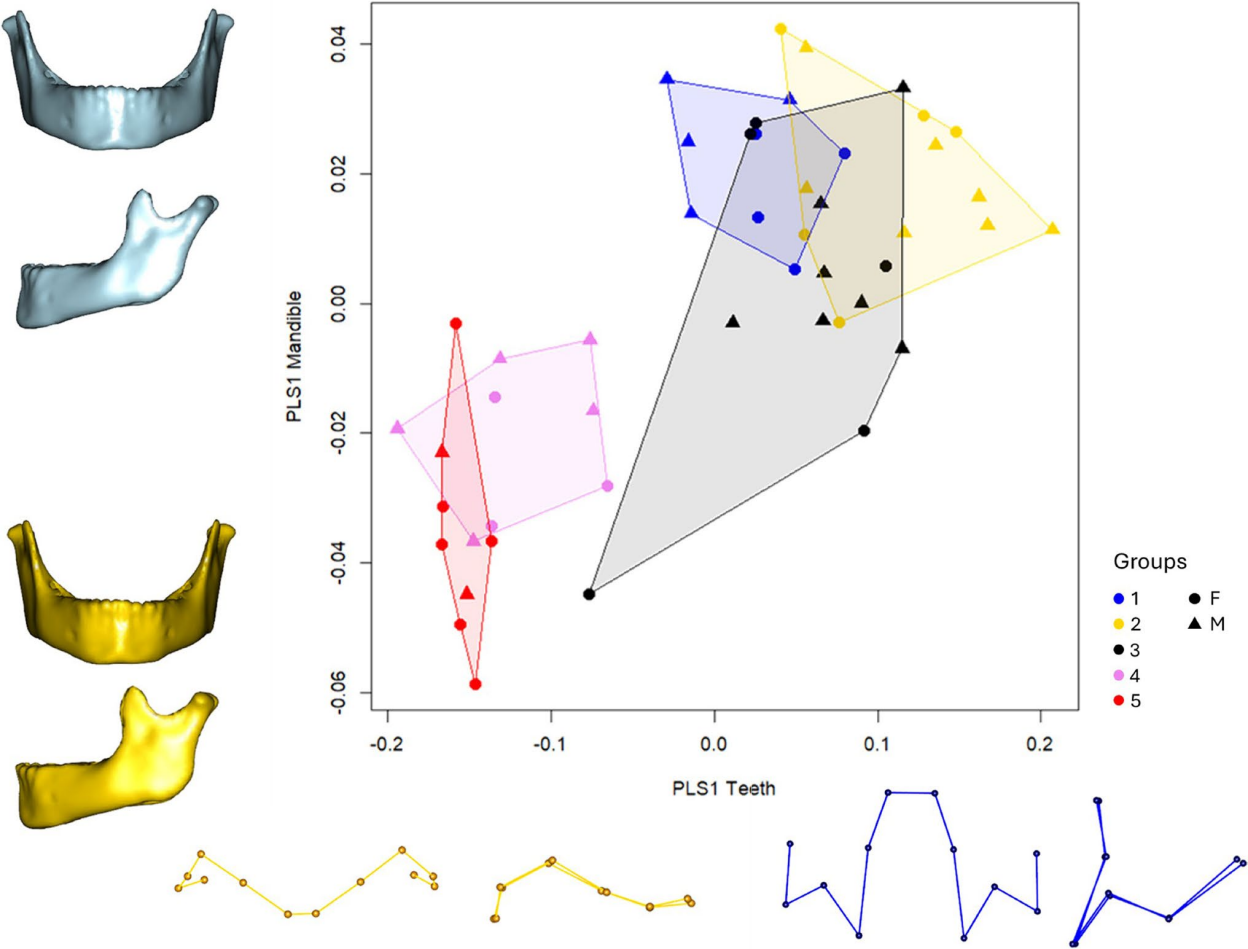
Two‐block partial least squares on T configuration (PLS1) vs. M configuration (PLS2). Shape variations are shown along the correspondent axis: M configurations are represented in frontal (top) and lateral (bottom) views, while T configurations are shown in frontal (right) and left lateral (left) views. Yellow, minimum value; Blue, maximum value (G1: 48–61 months; G2: 53–91 months; G3: 84–126 months; G4: 99–148 months; G5: 134–155 months).

To examine the covariation between T and M configurations, we applied a PLS analysis to the Procrustes coordinates. The results, presented in Figure 6, prove a strong relationship between the configurations (*R* ^2^ = 0.287, *p* = 0.000999). PLS 1, associated with the T configuration, illustrates the transition from a state where all teeth are unerupted (maximum values) to fully erupted teeth (minimum value). In contrast, PLS 2, reflecting mandibular shape, captures changes from maximum to minimum values, including a reduction in the mandibular angle, an increase in mandibular corpus height, and greater ramus width, among other features. In this morphospace, G1, G2, and G3 are clustered in the maximum values for both configurations, while G4 and G5 align with the minimum values.

Overall, inter‐group variability appears reduced compared to the PCA results, with G2 and G3 exhibiting the most notable variation. Among these, G3 stands out as the group with the greatest variability and represents an intermediate shape in both M and T configurations.

Interestingly, one individual (Table S1) from G3 was identified as an outlier, clustering more closely with G4 and G5 in the analysis. Upon reviewing the chronological age (123 months), it was found that this individual is older than the other members of G3, and more closely resembles the female individuals from G4 in terms of chronological age.

### 3.4 Linear Regression

**FIGURE 7:**
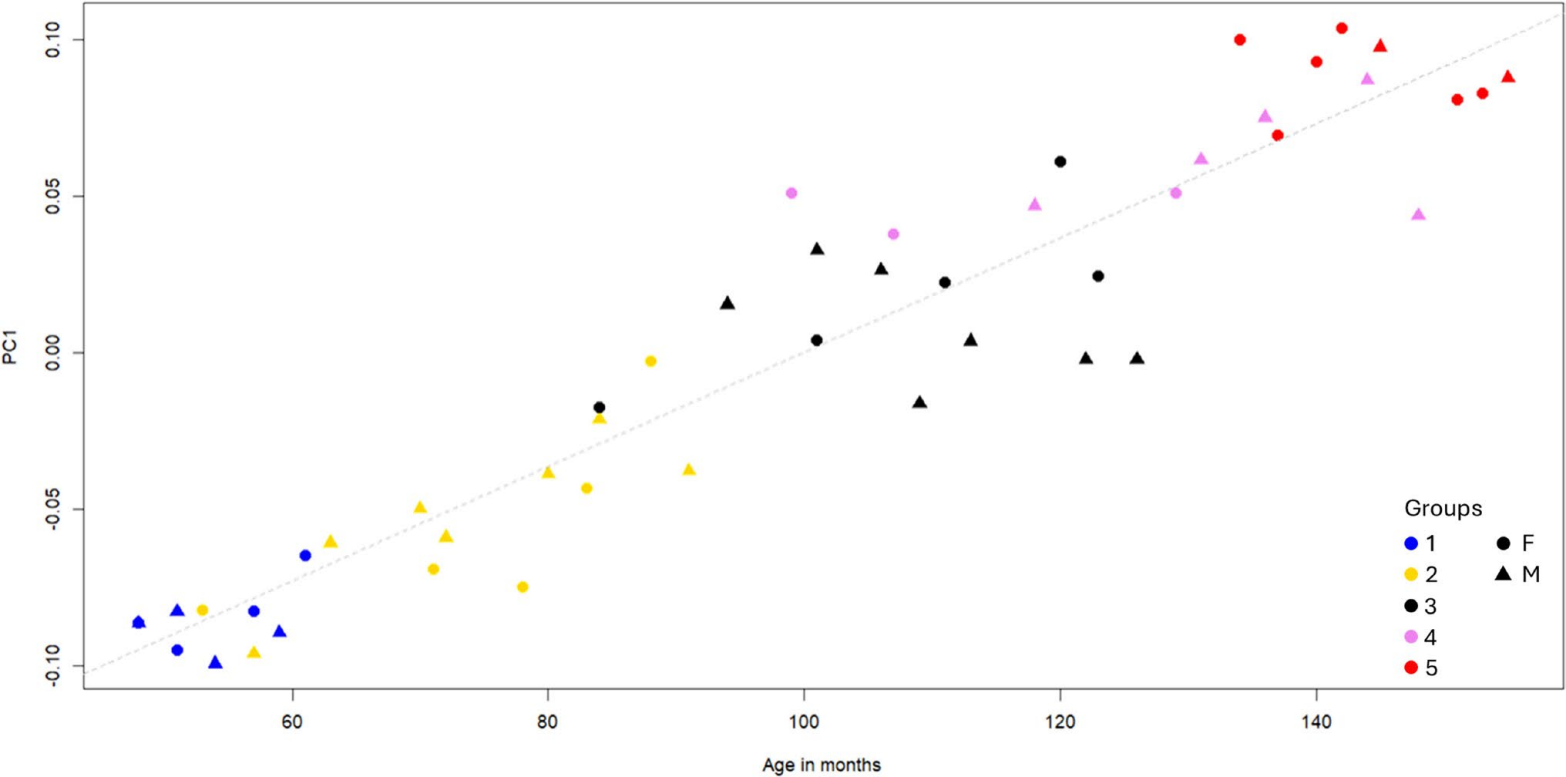
Regression of Principal Component 1 of the TM configuration against the chronological age of the individual, *R* ^2^ = 0.8936, *p* < 2.2e−16. (G1: 48–61 months; G2: 53–91 months; G3: 84–126 months; G4: 99–148 months; G5: 134–155 months).

To evaluate the effectiveness of our proxies for age estimation, we performed linear regression analyses on the PC scores of both separate and combined configurations against the sample's chronological age in months. The results indicate a low coefficient of determination for the individual configurations: *R* ^2^ = 0.46 for the M configuration and *R* ^2^ = 0.23 for the T configuration (Figures S1 and S2). In the latter case, the poor fit suggests a polynomial regression, likely due to the high variability in eruption patterns. Biologically, this can be explained by the fact that in G1 and G4–5, tooth cusps are at a similar vertical height—either not yet erupted (G1) or fully erupted (G4–5)—whereas in G2–3, teeth are at various stages of eruption, leading to greater variation (Figure S2). However, when the configurations were combined (TM), the model fit improved significantly, reaching *R* ^2^ = 0.89 (Figure 7).

## 4 Discussion

This study aims to investigate the relationship between mandibular shape, dental eruption patterns, and chronological age. We used two landmark configurations, teeth and mandibular shape, which were assessed separately (T and M) and combined (TM).

### 4.1 Separated Configuration

The results from the separate configurations provided valuable insights into the variation of each proxy within the groups considered, offering distinct yet meaningful information. Specifically, the T configuration yielded expected results, as groups aligned with dental eruption stages (Figure 4). However, unlike the TM PCA (Figure 2), the groups in the T PCA did not display a clear sequential distribution. Instead, they followed a circular clockwise trajectory, starting with G1 at the top and ending with G5 on the left, and showed greater variability within each group, with the exception of G5. This suggests that dental eruption is a highly variable proxy, particularly during the mixed dentition period, likely influenced by various environmental and genetic factors (Hillson 2005; Almonaitiene et al. 2010). Studies on twins and family lineages have confirmed a strong heritable component in eruption timing which in turn causes variation among different populations (Tompkins 1996; Hughes et al. 2007; Bockmann et al. 2010). Variation within a population is likewise abundant, especially in the eruption of canines and premolars (Smith and Garn 1987). Moreover, nutritional stress, epigenetic mechanisms in early childhood, results in malnutrition and chronic illness in childhood have been associated with delayed eruption (Kreiborg and Jensen 2018).

In contrast, the PCA based solely on mandibular shape (M configuration) revealed lower values for the most explanatory PC scores and a substantial amount of intra‐group variation, with considerable overlap observed (Figure 5). This outcome implies that mandibular shape variation alone may not be sufficient to account for the observed inter‐group differences, supporting similar conclusions in previous studies that questioned the effectiveness of mandibular shape as a standalone proxy for age estimation (Franklin and Cardini 2007; Franklin et al. 2008). Indeed, mandibular growth is more variable in juveniles and subadults compared to adults and is influenced by the development of the dentition, as well as the biomechanical stresses of respiration, chewing, swallowing, and speech (Freidline et al. 2017; Humphrey 1998; Goose and Appleton 1982). Furthermore, the patterns of changes in mandibular morphology due to dental development are not stable and are inherently dynamic, contributing to increased variability (Polanski 2011).

### 4.2 Identification of the Group‐Specific Trends in the Combined Configuration

Considering the combined configuration (TM) our analysis highlighted distinct trends among different age groups. Along PC1 (41.93%), it provided a clear separation among the groups ranging sequentially from G1 to G5; along PC2 (8.52%), it showed the effect of the eruption of the first molar, lateral and central incisors, with the mandibular shape variation not visibly affecting the group variability. G1 and G5 had the smallest inter‐individual variability while G2 showed the largest variation, particularly along PC2. Moreover, groups with higher variation had higher PC2 scores and vice versa.

We hypothesize that individuals from G2, G3, and G4, who are in the stage of the eruption of the first molar, with central and lateral incisors reaching the alveolar level (partial eruption), do not exhibit significant mandibular variation until the eruption of canine and premolars. Generally, canine and premolars erupt within 10–13 years of age (Liversidge 2015; Ubelaker 1987; White et al. 2011). This time frame also corresponds to the pubertal growth spurt, which usually occurs at 13 years of age in males and 10 years in females (Cameron 2022). The mandible has a period of minimal growth velocity immediately before the onset of the adolescent growth spurt, also known as prepuberal minimum or adrenarche (Franchi et al. 2001). This results in the upward‐forward direction of condylar growth, and reduction of the gonial angle along with the upward‐forward direction of growth at the condyle (Scheuer and Black 2000; Krarup et al. 2005; Hutchinson et al. 2012). Studies have also shown that the eruption of the incisors and canine has a significant impact on the upward‐backwards direction of growth at the symphysis (Tsai 2004; Krarup et al. 2005; Franklin and Cardini 2007; Fukase and Suwa 2010; Coquerelle et al. 2011, 2013; Hutchinson et al. 2012; Zimmer et al. 2023).

Interestingly, the investigation of mandibular shape and tooth configuration using PLS analysis provided valuable insights into group variability. The strong relationship observed between the two configurations is expected, as mandibular growth is closely influenced by tooth eruption (Figure 6) (Šešelj et al. 2019). However, the clear separation between groups G1, G2, and G3 on one side, and G4 and G5 on the other, is particularly notable. This distinct separation suggests a significant morphological shift in both mandibular shape and tooth configuration at this stage, which may be associated with the growth spurt. This interpretation is further reinforced by the centroid size of the mandibular configuration, which shows a marked increase from G3 onward (Figure 3). Interestingly, G3 and G4 correspond to chronological age ranging from 8 to 12.3 years old according to the sample analysis, which matches the period of puberty in both sexes.

Although the investigation of growth spurts and associated sexual dimorphism was not the primary focus of this study, we did not observe significant differences between males and females in our sample. These findings align with previous research on mandibular sexual dimorphism, which reported that morphological differences between sexes in individuals aged 4 to 14 years are not readily discernible (Coquerelle et al. 2011). In this context, we hypothesize that the morphological variations identified in groups G3 and G4 represent stages around the pubertal growth spurt, independently of the sex.

However, the considerable variability within these groups might be attributed to the wide range of developmental timing among individuals. Further studies with a larger sample size and smaller age range could help clarify these developmental stages and better account for the variability observed in these groups.

### 4.3 Testing the Estimation of Chronological Age

From the results of the linear regression, it is evident that the combined configuration provides a robust proxy for improving age‐at‐death estimation of mandibular remains. Increasing the sample size and narrowing the chronological age range of analyzed individuals could enhance the resolution of this approach and facilitate the development of a standardized protocol for a more precise chronological age estimation.

## 5 Conclusions

In this study, geometric morphometrics enabled us to quantitatively analyze subtle changes in the mandibular complex during dental development in juveniles, changes that are otherwise challenging to quantify in skeletal samples. Specifically, our analysis not only identified group‐specific trends among individuals during phases of dental eruption but also pinpointed a strong association between phases of dental formation and mandibular growth within a narrow age bracket (3 to 6 months). Compared to the performance of the two proxies analyzed separately (dental eruption pattern and mandibular shape), the combined configuration captured a significant portion of the overall variation present in the dataset.

Mandibular shape variation is most pronounced in the youngest groups and decreases as growth progresses. When considered together with stages of dental eruption, this pattern allows for the identification of precise periods of morphological change. Indeed, coupling mandibular variation and dental eruption stages through PLS allowed us to observe a critical morphological change between 8 and 12.3 years old according to the sample analyzed, matching the growth spurt in modern humans. Finally, the linear regression combining the results of the PCA on both configurations against the chronological age showed a strong fit, offering promising possibilities for standardized age‐at‐death estimation protocols for bioarchaeology or even paleoanthropological samples.

## Author Contributions

**Tannistha Chakraborty:** conceptualization (equal), data curation (equal), formal analysis (equal), investigation (equal), methodology (equal), resources (equal), software (equal), validation (equal), visualization (equal), writing – original draft (equal), writing – review and editing (equal). **Julie Arnaud:** conceptualization (equal), data curation (equal), formal analysis (equal), investigation (equal), methodology (equal), supervision (equal), validation (equal), visualization (equal), writing – original draft (equal), writing – review and editing (equal). **Costantino Buzi:** conceptualization (equal), data curation (equal), formal analysis (equal), methodology (equal), software (equal), supervision (equal), validation (equal), visualization (equal), writing – original draft (equal), writing – review and editing (equal).

## Conflicts of Interest

The authors declare no conflicts of interest.

## Supporting information


**Data S1:** ajpa70155‐sup‐0001‐Supinfo.docx.

## Data Availability

The full R codes and data used for the present study are available on Zenodo (10.5281/zenodo.14706526).
