# Supplementary material for: Exploring the Relationship Between Mandibular Morphology, Dental Eruption, and Chronological Age in Modern Human Juveniles Through Geometric Morphometrics
Source: Am J Biol Anthropol. 2025 Nov 12;188(3):e70155. doi: 10.1002/ajpa.70155 (PMC12607250; doi:10.1002/ajpa.70155)
Supplement: Supplementary file 1 — Data S1: ajpa70155‐sup‐0001‐Supinfo.docx. [file AJPA-188-e70155-s001.docx]

**Table S1.** Sample list categorised according to the classification by Liversidge & Molleson (2004), including the observed stage of each tooth in the individuals. G1: teeth unerupted; G2: partial eruption of molar and incisors; G3: complete eruption of molars and incisors; G4: partial eruption of premolars and canine; G5: complete eruption. U: unerupted; AE: alveolar eruption; PE: partial eruption; E: complete/occlusal eruption; R: right side; L: left side.

| Group | Age (months) | Sex | M1 | P2 | P1 | C | I2 | I1 |
| --- | --- | --- | --- | --- | --- | --- | --- | --- |
| G1 | 48 | F | U | U | U | U | U | U |
|  | 51 | F | U | U | U | U | U | U |
|  | 57 | F | U | U | U | U | U | U |
|  | 61 | F | U | U | U | U | U | U |
|  | 48 | M | U | U | U | U | U | U |
|  | 51 | M | U | U | U | U | U | U |
|  | 54 | M | U | U | U | U | U | U |
|  | 59 | M | U | U | U | U | U | U |
| G2 | 53 | F | AE | U | U | U | U | U |
|  | 71 | F | AE | U | U | U | U | U |
|  | 78 | F | PE | U | U | U | U | U |
|  | 83 | F | E | U | U | U | AE | E |
|  | 88 | F | E | U | U | U | E | E |
|  | 57 | M | AE | U | U | U | U | U |
|  | 63 | M | E | U | U | U | U | U(L)/AE(R) |
|  | 70 | M | AE | U | U | U | U | U |
|  | 72 | M | AE | U | U | U | PE | PE |
|  | 80 | M | E | U | U | U | U | E |
|  | 84 | M | PE | U | U | U | U | E |
|  | 91 | M | E | U | U | U | U | E |
| G3 | 84 | F | E | U | U | U | E | E |
|  | 101 | F | E | U | U | U | E | E |
|  | 111 | F | E | U | U | U | E | E |
|  | 120 | F | E | U | U | U | E | E |
|  | 94 | M | E | U | U | U | E | E |
|  | 101 | M | E | U | U | U | E | E |
|  | 106 | M | E | U | U | U | E | E |
|  | 109 | M | E | U | U | U | E | E |
|  | 113 | M | E | U | U | U | E | E |
|  | 122 | M | E | U | U | U | E | E |
|  | 123 | F | E | U | U | U | E | E |
|  | 126 | M | E | U | U | U | E | E |
| G4 | 99 | F | E | U | U | E | E | E |
|  | 107 | F | E | U | PE(L)/E(R) | PE | E | E |
|  | 129 | F | E | U | E | E | E | E |
|  | 118 | M | E | U | U | R(AE)/L (U) | E | E |
|  | 131 | M | E | PE(L)/E(R) | E | PE | E | E |
|  | 136 | M | E | U | E | E | E | E |
|  | 144 | M | E | U | E | E | E | E |
|  | 148 | M | E | U | E | E | E | E |
| G5 | 134 | F | E | E | E | E | E | E |
|  | 137 | F | E | E | E | E | E | E |
|  | 140 | F | E | E | E | E | E | E |
|  | 142 | F | E | E | E | E | E | E |
|  | 151 | F | E | E | E | E | E | E |
|  | 153 | F | E | E | E | E | E | E |
|  | 145 | M | E | E | E | E | E | E |
|  | 155 | M | E | E | E | E | E | E |

**Table S2.** Landmark configuration used for the dental eruption pattern. The points indicated with * are bilateral and have to be considered as both left and right

| N° | Point | Type | Description |
| --- | --- | --- | --- |
| *1, 7* | M_1_* | II | Tip of the mesiobuccal cusp (i.e. protoconid) |
| *2, 8* | P_4_* | II | Tip of the buccal cusp |
| *3, 9* | P_3_* | II | Tip of the buccal cusp |
| *4, 10* | C_,_* | II | Tip of the cusp |
| *5, 11* | I_2_* | III | Midpoint of the incisal margin |
| *6, 12* | I_1_* | III | Midpoint of the incisal margin |

**Table S3.** Landmark configuration used for the mandible. The points indicated with * are bilateral and have to be considered as both left and right; § refers to points defined in Bastir & Rosas (2004); ‡ refers to points defined in Rosas (2002).

| N° | Point | Type | Description |
| --- | --- | --- | --- |
| *1, 13* | Canine | II | The mesial point of the canine crown on the alveolar border |
| *2, 14* | Mental foramen* | II | Foramen located on the lateral corpus surface, near mid-corpus, below the premolar region |
| *3, 15* | Inferior basal border* | II | Posterior beginning of the inferior basal border |
| *4, 16* | Pre-angular notch* | II | The notch on the inferior basal border before the gonion |
| *5, 17* | Gonion* | I | Point along the rounded posteroinferior corner of the mandible between the ramus and the body |
| *6, 18* | Ramus flexure* | II | Concave mandibular notch that separates the condyle and coronoid process |
| *7, 19* | Condylion* | II | The most medial point on the right mandibular condyle |
| *8, 20* | Mandibular notch* | II | Maximum flexion of curvature § |
| *9, 21* | Coronoid process* (Right, Left) | II | Anterior-superior tip of coronoid process § |
| *10, 22* | Anterior ramus* | II | Anterior point of minimal ramus breadth § |
| *11, 23* | Posterior alveolar process end* | II | End of superior alveolar border and ramus process at the end of dental arcade |
| *12, 24* | Mandibular foramen* | II | the most inferior point on the margin of the mandibular mental foramen |
| *25* | Infradentale | I | midline point at the superior tip of the septum between the mandibular central incisor |
| *26* | B-point | II | The deepest point at the mandibular symphysis curvature ‡ |
| *27* | Menton | I | the triangular eminence, or bony chin, at the base of the corpus in the anterior symphyseal region |
| *28* | Gnathion | II | most inferior midline points on the mandible. |
| *29* | Foramen genioglossus | I | Foramen for genioglossus muscle in the inner surface of the mandible |
| *30* | Internal infradentale | I | The midline points at the superior tip of the septum between the mandibular central incisor on the inner surface of the mandible |

**Table S4. By group variance for PC1 and PC2, combined configuration.** A Brown-Forsyte test was first performed to assess the variances between groups and within groups. The results for the separate PCs are reported below, alongside the R code used to perform the analysis and compute the variance by group.

| Group | PC1 | PC2 |
| --- | --- | --- |
| G1 | 0.0001063888 | 0.0001579107 |
| G2 | 0.0006921453 | 0.0009893516 |
| G3 | 0.0005000187 | 0.0001596152 |
| G4 | 0.0002786386 | 0.0005564918 |
| G5 | 0.0001288108 | 0.0001082226 |

**Brown-Forsythe Test (PC1).**

data: PC1 and Group

F = 139.38; num df = 4.000; denom df = 36.615; p-value < 2.2e-16

**Brown-Forsythe Test (PC2).**

data: PC2 and Group

F = 14.505; num df = 4.00; denom df = 27.93; p-value = 1.588e-06

R functions used: functions *bf.test()*, package "onewaytests", Dag et al., 2018, and *aggregate()*, package "stats".

Dag, O., Dolgun, A., Konar, N.M. (2018). onewaytests: An R Package for One-Way Tests in Independent Groups Designs. The R Journal, 10(1), 175-199.

**R code used for the analysis:**

# Create dataframe

Var_TM**<-**data.frame**(**Group**=**GRO,PC1**=**pca**$**PCscores**[**,1**]**,PC2**=**pca**$**PCscores**[**,2**])**

# Brown-Forsythe test (PC1)

library**(**onewaytests**)**

bf_res**<-**bf.test**(**PC1**~**Group,data**=**Var_TM**)**

print**(**bf_res**)**

# Brown-Forsythe test (PC2)

bf_res_2**<-** bf.test**(**PC2**~**Group,data**=**Var_TM**)**

print**(**bf_res_2**)**

# Variance by group (PC1)

VBG**<-**aggregate**(**PC1**~**Group,data**=**Var_TM,FUN**=**var**)**

print**(**VBG**)**

# Variance by group (PC2)

VBG_2**<-**aggregate**(**PC2**~**Group,data**=**Var_TM,FUN**=**var**)**

print**(**VBG_2**)**


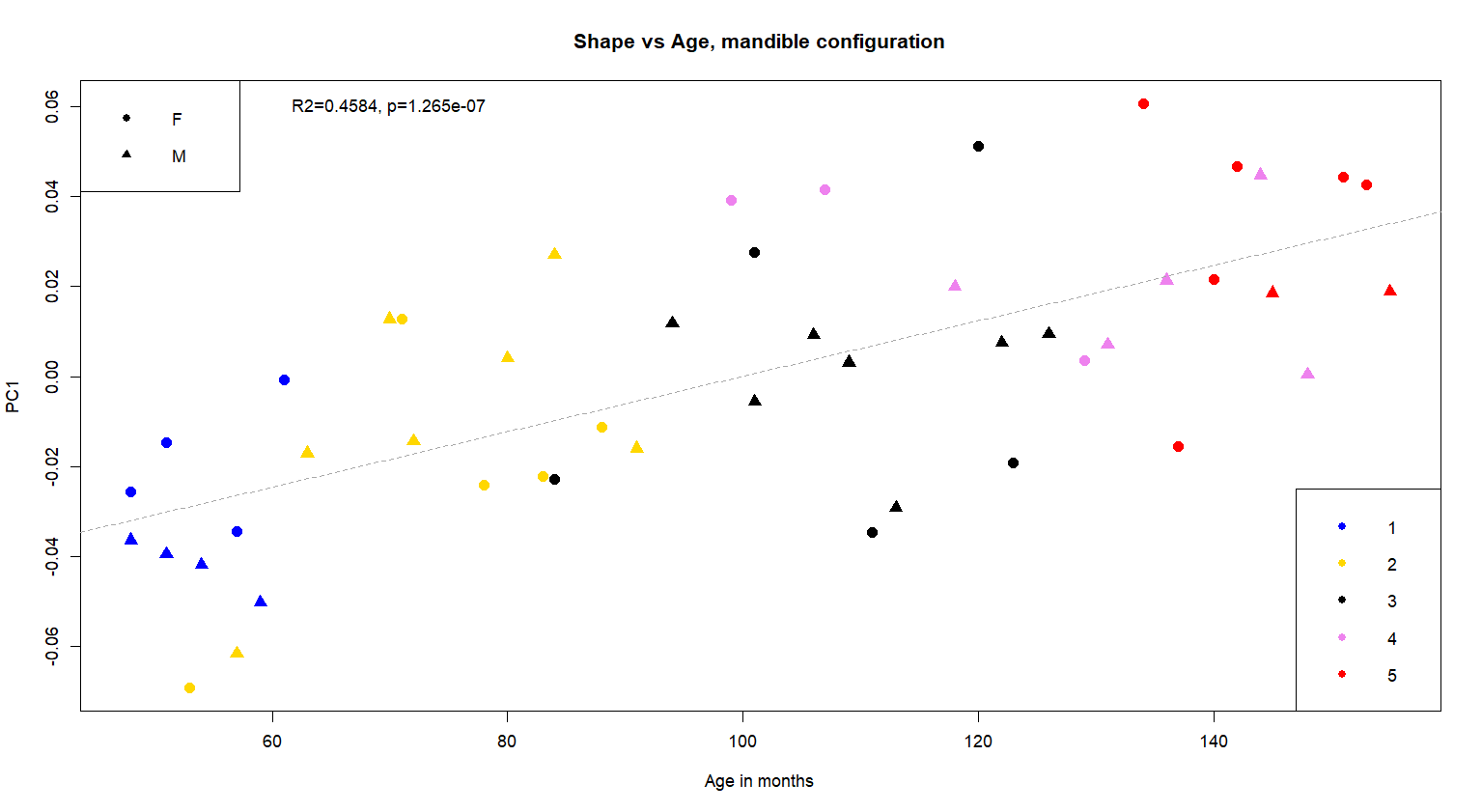
 **Figure S1.** Regression of PC1 of the mandibular configuration against the chronological age of the individual.
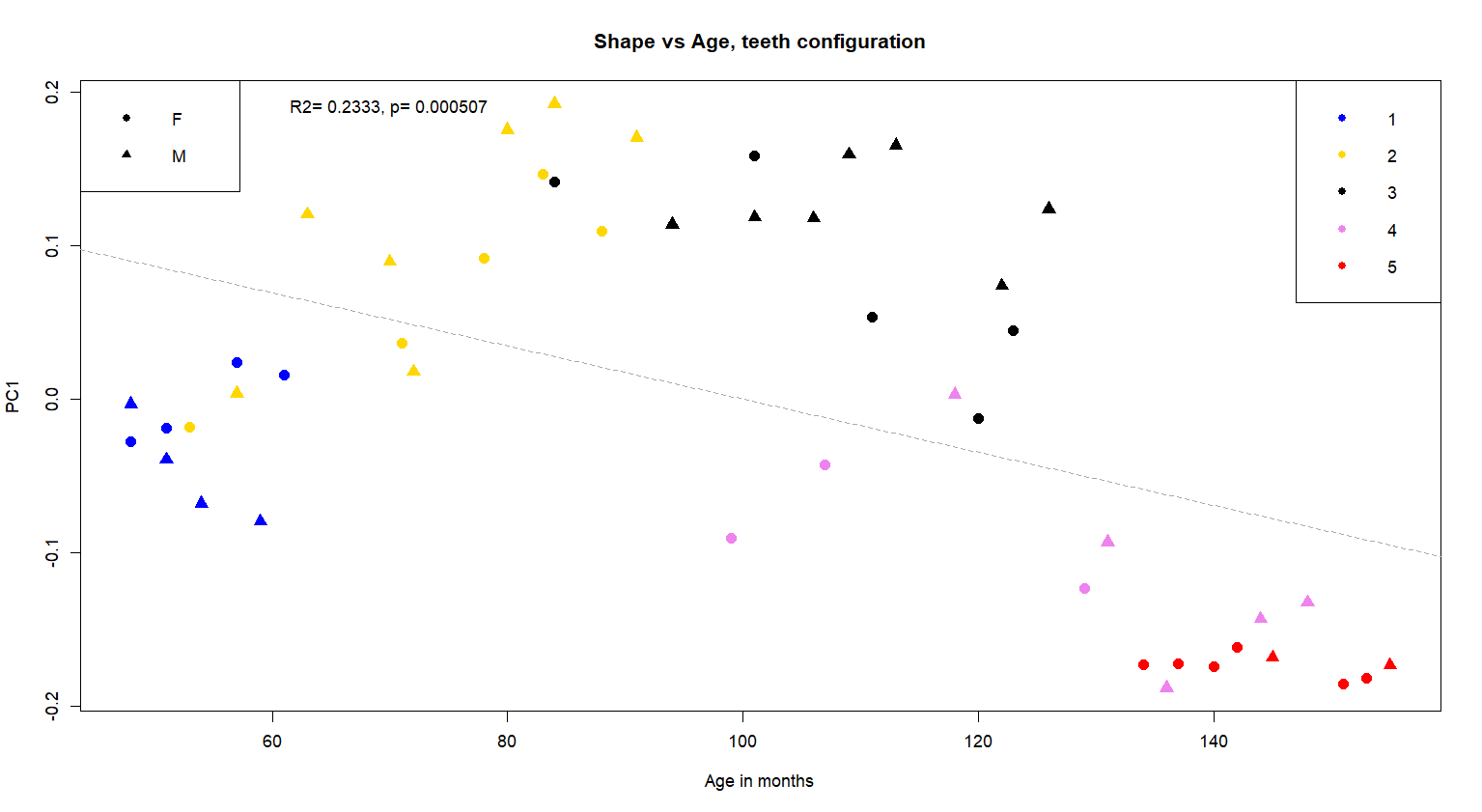


**Figure S2.** Regression of PC1 of the teeth configuration against the chronological age of the individual.
